# On the Influence of AQM on Serialization of Packet Losses

**DOI:** 10.3390/s23042197

**Published:** 2023-02-15

**Authors:** Andrzej Chydzinski, Blazej Adamczyk

**Affiliations:** Department of Computer Networks and Systems, Silesian University of Technology, Akademicka 16, 44-100 Gliwice, Poland

**Keywords:** packet networks, active queue management, burst ratio, serial losses

## Abstract

We study the influence of the active queue management mechanism based on the queue size on the serialization of packet losses, i.e., the occurrences of losses in long, consecutive series. We use a traffic model able to mimic precisely the autocorrelation function of traffic, which is known to be far from zero in packet networks. The main contribution is a theorem on the burst ratio parameter, describing the serialization of losses, proven for an arbitrary function assigning drop probabilities to queue sizes. In numerical examples, we show the impact of the autocorrelation strength, drop probability function, and load of the link, on the serialization of losses.

## 1. Introduction

In TCP/IP networks, some packets are deleted in network nodes (routers). This is connected to the fundamental design principle of most such networks: no resource reservation is needed before sending any volume of data, but no guarantee about the delivery is given. Therefore, the rate of traffic aggregated from a few input links may occasionally exceed the capacity of an output link. If this happens, the buffer of the output link becomes full and newly arriving packets are deleted, at least during some period of time.

As widely discussed in the literature, such a simple loss mechanism has several drawbacks. Among other things, it increases transmission delays, causes unfair bandwidth allocation between flows, and synchronizes the TCP control mechanisms of the hosts [1]. All of these are caused by the bufferbloat effect [2,3], i.e., frequent overfilling of packet buffers in networking devices.

Therefore, the Internet Engineering Task Force (IETF) recommends the deployment of active queue management (AQM) in routers and switches [1]. Generally, AQM assumes that packets can be dropped much earlier, when the buffer is far from being overflowed. Moreover, an arriving packet is dropped randomly, with some probability evolving in time. The IETF does not recommend just one algorithm of this type: many solutions have been proposed and tested to date, for instance [4,5,6,7,8,9].

Active queue management is also proposed for wireless sensor networks (WSNs) [10,11,12,13,14]). It can be especially useful in some sensitive applications of WSNs, such as healthcare [13]. Obviously, avoiding link congestions and packet losses is very important in such applications.

An important family of AQM algorithms uses the concept that the probability of deleting a packet should depend on the queue size. Many different drop probability functions have been considered to date, e.g., linear, quadratic, cubic, composed of linear and cubic, composed of linear and logarithmic, or beta (see [15,16,17,18,19,20], respectively). Recently, such AQM algorithms were implemented in a high-speed networking device and tested in an operating network of the campus of a large university [21].

The first, obvious characteristic of the packet loss process is the loss ratio, *L*. It has been widely studied using mathematical, simulation, and experimental methods. Therefore, we did not deal with it here.

The second important characteristic is the burst ratio, *B*. It is defined in Section 3, but for now, it is important to say that *B* characterizes the inclination of packet losses to occur one after another, in series. When B=1, the losses have neither the inclination to occur separately, nor in series: short series occur occasionally due to the random grouping of losses. When B>1, the losses have the inclination to occur in series. The larger *B* is, the stronger this inclination is. Finally, when B<1, the losses have the inclination to occur separately.

The importance of the burst ratio is connected to the fact that elevated values of *B* may seriously worsen any real-time transmission of the data, in a network of any type. In general, packet losses do not constitute a critical obstacle in real-time transmissions. The well-known redundant coding enables tolerance to packet losses, by allowing the recreation of missing information from successfully delivered packets. Unfortunately, this becomes difficult or impossible when the losses tend to occur in long, consecutive series. A well-studied case is the real-time transmission of packetized voice, for which a formula was developed that reflects the deterioration of the quality of voice transmissions as a function of *B* (see page 8 of [22]). The reasoning, however, is the same for any type of real-time transmission, not necessarily voice or even multimedia. In a WSN, we may want to transmit other types of information from sensors, in real-time. The redundant coding can prevent the transmission due to the loss of information due to the packet losses, but the higher the value of *B* is, the less effective this becomes.

In the experimental study of [21], two things were demonstrated about the burst ratio. Firstly, when no AQM is used, the burst ratio is always greater than 1. Secondly, the application of AQM reduces its value substantially.

In this paper, we studied this effect, i.e., how and to what extent an AQM based on the queue size reduces the burst ratio, using a mathematical model of AQM. This has not yet been performed with a complex, autocorrelated model of traffic. It is well known that packet interarrival times in TCP/IP networks are positively autocorrelated [23,24]. It is also known that this autocorrelation has a profound influence on the queueing performance characteristics.

Therefore, the two main goals of this paper were:

(i) To derive the burst ratio in a model of the system with AQM based on the queue size and the arrival process such that it enables arbitrary modeling of the autocorrelation function and arbitrary modeling of the distribution of the interarrival time;
(ii) To present sample results for different system parameterizations, in which we can see the influence of the autocorrelation, as well as other system characteristics on the burst ratio.

The most-important contribution of the paper is Theorem 1, in which a formula for the burst ratio in an AQM queue fed by the Markov-modulated Poisson process (MMPP) is proven. The MMPP is known to fulfill the requirements stated in Goal (i): it indeed enables arbitrary modeling of the autocorrelation function, as well as the interarrival time distribution [25]. Theorem 1 is general in the sense that the drop probability function is not specified there; it may assume an arbitrary form.

Goal (ii) was realized by presenting and discussing the numerical results for a family of arrival processes, in which the strength of the autocorrelation can be controlled by a parameter, and for a family of drop probability functions, in which the strength of the AQM can be controlled by another parameter. Based on these results, several interesting observations on the values of *B* under different networking conditions were made.

The remaining sections of the paper are organized in the following way. In Section 2, we recall previous analytical work either on the burst ratio or on queuing models of AQM based on the queue size. In Section 3, we define the queueing model of interest and recall the definitions of the MMPP and the burst ratio. In Section 4, the main theorem on the burst ratio is presented and proven. Then, in Section 5, the numerical results are shown and discussed. They include the burst ratios computed for variable autocorrelations, drop probability functions, and link loads. In addition, the simulation results are presented for the verification of the theoretical results. In Section 6, the results of the paper are recapped.

## 2. Previous Work

To the best of the authors’ knowledge, the results presented here are new.

The burst ratio as a measure of the serialization of packet losses was proposed in [26]. Then, it was studied in [26,27,28,29,30], using various models of losses. None of these works, however, included an AQM mechanism.

On the other hand, queueing models of systems with AQM based on the queue size were studied in [31,32,33,34,35,36,37,38,39]. In these papers, however, only classic queueing characteristics were derived, including the queue size distribution and the loss ratio. In none of them was the burst ratio of packet losses studied.

Finally, in two articles, the burst ratio in a queue with the AQM mechanism was investigated [40,41]. However, in both of them, the arrival process was a simple renewal process with zero autocorrelation. In real networks, the autocorrelation is far from zero. Moreover, it is known that autocorrelation has a deep impact on the queueing performance characteristics, which could not be studied in models of [40,41].

## 3. Queueing Model and Notations

We dealt with the single-server queue enhanced by the AQM mechanism based on the queue size and fed by an autocorrelated traffic.

Namely, packets arrive according to the MMPP (defined below) at a buffer of size *K* packets, where they form a queue. This queue is served by an output link. The service time distribution of a packet is exponential with parameter μ.

The AQM mechanism utilizes a predefined drop probability function d(n). Namely, when a packet arrives, there are *n* packets in the buffer, the new packet is put into the buffer or deleted with probabilities 1−d(n) and d(n), respectively. Moreover, a new packet is always deleted if, upon its arrival, the buffer is full. This is equivalent to assuming that d(n)=1 if n≥K, except that function d(n) may assume any form.

To define the Markov-modulated Poisson process [42], we need a continuous-time Markov chain J(t) with states {1,…,m} and infinitesimal matrix *Q*. Moreover, we need vector $\left[\lambda_{1},\ldots ,\lambda_{m}\right]$. The temporary behavior of the MMPP is that of the Poisson process at rate $\lambda_{J(t)}$. In other words, the evolution of the Markov chain determines the rate of Poisson arrivals. In practice, we often use rates $\left[\lambda_{1},\ldots ,\lambda_{m}\right]$ in the form of a diagonal matrix:

$$\begin{array}{cc} \Lambda & =\mathrm{diag}[\lambda_{1},\ldots ,\lambda_{m}]. \end{array}$$

The basic properties and characteristics of the MMPP are known and can be found in [42]. In this paper, the k−lag autocorrelation of interarrival times in the MMPP is especially important, which can be found on page 153 of [42]. We also need the total rate of the MMPP, λ (see Formula (10) below). As was already said, the MMPP enables fitting the autocorrelation function and the interarrival time distribution to observed traffic very well [25].

The load of the link is defined as usual, $\rho =\frac{\lambda }{\mu }$. By X(t), we denote the queue size at time *t*. It was assumed that the packet under service is included in X(t), if applicable. Furthermore, *K* includes the service position as well. We used the convention that random process X(t) is left-hand side continuous, i.e., X(t−)=X(t) for every *t*.

Finally, we may define the burst ratio characteristic, *B*, following [26]. Given a long sequence of packets, some of them accepted into the buffer and some of them lost upon arrival, we have:

(1) $$B=\frac{\bar{G}}{\bar{K}},$$

where $\bar{G}$ is the mean length of the series of lost packets in the studied sequence, while $\bar{K}$ is the theoretical mean length of the series of lost packets, expected in the case when all the losses are random and independent of each other.

If the loss ratio in the considered sequence is *L*, then a simple verification shows that $\bar{K}=1/\left(1-L\right)$. Therefore, we have another, more useful formula for the burst ratio:

(2) $$B=\bar{G}\left(1-L\right).$$

Consider the following sequence: + + + – – + + + + – – – + + + +, where “+” denotes a packet accepted into the buffer, while “–” denotes a lost packet. The mean length of the series of lost packets is $\bar{G}=\left(2+3\right)/2=2.5$, while the loss ratio is L=(2+3)/16=0.3125. Hence, the theoretical mean length of the series, expected in the case when all the losses are random and independent of each other, is $\bar{K}=1/\left(1-0.3125\right)=1.4545$. Finally, B=2/1.45=1.38. B>1 means that the losses in the sequence have an inclination to occur in series. This inclination is not very strong in the analyzed sequence; perhaps we would have said that if it was B>2.

## 4. Analysis

Before we prove the main theorem of the burst ratio, it is necessary to find the stationary joint distribution of the queue size and the modulating state. Firstly, note that (X(t),J(t)) is a continuous-time Markov chain. It is easily seen that its generator matrix, *P*, has the following form:

(3) $$P=\left(\begin{array}{cccccc} A & D_{0} & 0 & 0 & \cdots & 0\\ \mu I & C_{1} & D_{1} & 0 & \cdots & 0\\ 0 & \mu I & C_{2} & D_{3} & \cdots & 0\\ 0 & 0 & \mu I & C_{3} & \ddots & 0\\ \vdots & \vdots & \vdots & \ddots & \ddots & \vdots \\ 0 & 0 & 0 & \cdots & \mu I & C_{K} \end{array}\right),$$

with

(4) A=Q−(1−d(0))Λ,

(5) $$C_{n}=Q-\left(1-d\left(n\right)\right)\Lambda -\mu I,$$

(6) $$D_{n}=\left(1-d\left(n\right)\right)\Lambda ,$$

where *I* denotes the m×m identity matrix. The stationary probabilities, i.e.,

(7) $$p_{n,i}=\lim_{t\to \infty }P\left\{X\left(t\right)=n,J\left(t\right)=i\right\},$$

can be obtained, as usual, by solving the system:

(8) $$pP=\left[0,\ldots ,0\right],\;\;\;\sum_{n=0}^{K}\sum_{i=1}^{m}p_{n,i}=1,$$

where

(9) $$p=\left[p_{1},\ldots ,p_{K}\right],\;\;\;\;p_{n}=\left[p_{n,1},\ldots ,p_{n,m}\right].$$

Having computed the vectors $p_{n}$, we can obtain the total arrival rate, λ, of the MMPP:

(10) $$\lambda =\sum_{n=0}^{K}p_{n}\Lambda e,$$

where e is a column vector of 1s. Obviously, λ can also be obtained in other ways as well. Herein, we used probabilities $p_{n}$ to compute λ, because they are needed for other purposes as well.

Now, we may prove the main result of the paper.

**Theorem** **1.** *The burst ratio of packet losses in a queue with MMPP arrivals and drop probabilities d(n) is equal to:*

(11) $$B=\frac{\left(1-L\right)\sum_{n=0}^{K}h_{n}E_{n}}{\sum_{n=0}^{K}h_{n}e},$$

*where*

(12) $$E_{0}={\left(I-d\left(0\right){\left(\Lambda -Q\right)}^{-1}\Lambda \right)}^{-1}e,$$

(13) $$\begin{array}{c} E_{n}={\left(I-d\left(n\right)M_{0}\right)}^{-1}\left(\sum_{k=1}^{n-1}d\left(n-k\right)M_{k}E_{n-k}+d\left(0\right){\left(\Lambda -Q\right)}^{-1}\Lambda E_{0}-d\left(0\right)\sum_{k=0}^{n-1}M_{k}E_{0}+e\right),\\ n>0, \end{array}$$

(14) $$h_{0}=\frac{d(0)}{\lambda }\sum_{k=0}^{K-1}\left(1-d(k)\right)p_{k}\Lambda \left({\left(\Lambda -Q\right)}^{-1}\Lambda -\sum_{u=0}^{k}M_{u}\right),$$

(15) $$h_{n}=\frac{d(n)}{\lambda }\sum_{k=n-1}^{K-1}\left(1-d(k)\right)p_{k}\Lambda M_{k+1-n},\;\;n>0,$$

(16) $$M_{k}=\frac{1}{k!}\int_{0}^{\infty }e^{-\mu t}{\left(\mu t\right)}^{k}\left(\Lambda -Q\right)e^{(Q-\Lambda )t}{\left(\Lambda -Q\right)}^{-1}\Lambda dt,$$

(17) $$L=\frac{1}{\lambda }\sum_{k=0}^{K}d\left(k\right)p_{k}\Lambda e,$$

*while λ and $p_{k}$ are given in (10) and (8), respectively.*

**Proof of Theorem 1.**  Denote by $T_{1}$, $T_{2},\ldots$ the successive arrival times and by $X_{1}$, $X_{2},\ldots$ the queue sizes at these arrival times, respectively. Define:

(18) $$F_{i,j}\left(t\right)=P\left\{T_{k+1}-T_{k}<t,J\left(T_{k+1}\right)=j|J\left(T_{k}\right)=i\right\},$$

In [42], it was shown that

(19) $$F\left(t\right)=\left(I-e^{(Q-\Lambda )t}\right){\left(\Lambda -Q\right)}^{-1}\Lambda ,$$

where

(20) $$F\left(t\right)={\left[F_{i,j}\left(t\right)\right]}_{i,j=1,\ldots ,m}.$$

From (19), we have

(21) $$f\left(t\right)=\left(\Lambda -Q\right)e^{(Q-\Lambda )t}{\left(\Lambda -Q\right)}^{-1}\Lambda ,$$

where

(22) $$f\left(t\right)={\left[f_{i,j}\left(t\right)\right]}_{i,j=1,\ldots ,m},\;\;\;f_{i,j}\left(t\right)=\frac{dF_{i,j}\left(t\right)}{dt}.$$

We first derived the mean number of losses from an arbitrary arrival time. Let us focus on some arrival time $T_{l}$, at which the queue size is *n* and the modulating state is *i*. Assume that the packet arriving at that time is lost, and let $E_{n,i}$ denote the mean number of losses from time $T_{l}$, including the one at $T_{l}$. If, at time $T_{l}$, the queue is not empty, then we can write the equation:

(23) $$\begin{array}{cc} E_{n,i}= & \sum_{k=0}^{n-1}\sum_{j=1}^{m}\int_{0}^{\infty }\frac{e^{-\mu t}{\left(\mu t\right)}^{k}}{k!}d\left(n-k\right)E_{n-k,j}f_{i,j}\left(t\right)dt\\ & +\sum_{k=n}^{\infty }\sum_{j=1}^{m}\int_{0}^{\infty }\frac{e^{-\mu t}{\left(\mu t\right)}^{k}}{k!}d\left(0\right)E_{0,j}f_{i,j}\left(t\right)dt+1,\;\;\;\;n=1,\ldots ,K. \end{array}$$

Namely, (23) is built conditioning on the state of the modulating chain upon the next arrival, *j*, and the duration of the interarrival time, $f_{i,j}\left(t\right)dt$. The first summand corresponds to the case where there are no more than n−1 service completions by time $T_{l+1}$ and the queue is not empty at $T_{l+1}$. The second summand corresponds to the case where there are *n* service completions by time $T_{l+1}$ and the queue becomes empty at $T_{l+1}$. The third summand corresponds to the loss at the time $T_{l}$.

Denote

(24) $$E_{n}={\left[E_{n,1},\ldots ,E_{n,m}\right]}^{T}.$$

From (23) and (21), we obtain:

(25) $$\begin{array}{c} E_{n}=\sum_{k=0}^{n-1}d\left(n-k\right)M_{k}E_{n-k}+d\left(0\right)\sum_{k=n}^{\infty }M_{k}E_{0}+e,\;\;\;\;n=1,\ldots ,K, \end{array}$$

where $M_{k}$ is defined in (16).

Assume now that the queue is empty at time $T_{l}$. In such a case, we see that it must hold that

(26) $$E_{0,i}=\sum_{j=1}^{m}d\left(0\right)a_{i,j}E_{0,j}+1,$$

where $a_{i,j}$ is the probability that the modulating chain changes its state from *i* to *j* during an interarrival time. From (18), we have:

(27) $$a_{i,j}=F_{i,j}\left(\infty \right).$$

Hence, from (19), it follows that $a_{i,j}$ is an (i,j) entry of the matrix ${\left(\Lambda -Q\right)}^{-1}\Lambda$. Using that, from (26), we obtain

(28) $$E_{0}=d\left(0\right){\left(\Lambda -Q\right)}^{-1}\Lambda E_{0}+e,$$

which finally leads to (12).

To simplify (25), we have

(29) $$\begin{array}{c} \sum_{k=n}^{\infty }M_{k}=\int_{0}^{\infty }\left(1-\sum_{k=0}^{n-1}\frac{e^{-\mu t}{\left(\mu t\right)}^{k}}{k!}\right)f\left(t\right)dt=\int_{0}^{\infty }f\left(t\right)dt-\sum_{k=0}^{n-1}M_{k}={\left(\Lambda -Q\right)}^{-1}\Lambda -\sum_{k=0}^{n-1}M_{k}. \end{array}$$

Hence, (25) and (29) yield:

(30) $$\begin{array}{c} E_{n}=\sum_{k=0}^{n-1}d\left(n-k\right)M_{k}E_{n-k}+d\left(0\right){\left(\Lambda -Q\right)}^{-1}\Lambda E_{0}-d\left(0\right)\sum_{k=0}^{n-1}M_{k}E_{0}+e,\;\;\;\;n=1,\ldots ,K. \end{array}$$

Finally, from (30), we obtain (13).

Define by $\pi_{n,i}$ the stationary probability of the queue size *n* and the modulating state *i* at an arrival epoch in the stationary regime, i.e.,

(31) $$\pi_{n,i}=\lim_{l\to \infty }P\left\{X_{l}=n,J\left(T_{l}\right)=i\right\},$$

and denote

(32) $$\pi_{n}=\left[\pi_{n,1},\ldots ,\pi_{n,m}\right].$$

(Note that $\pi_{n,i}$ differs from $p_{n,i}$ defined in (7)). We derive now the probability that a packet arriving at an arbitrary arrival time $T_{l}$ in the stationary regime is the one initiating a new series of losses. To achieve this, examine two consecutive arrival epochs: $T_{l-1}$, $T_{l}$. First, the probability of having arbitrary $X(T_{l-1})=k$ and $J(T_{l-1})=i$ is $\pi_{k,i}$. Second, the packet arriving at $T_{l-1}$ must be accepted, which happens with probability 1−d(k). Third, the transition from $X(T_{l-1})=k$, $J(T_{l-1})=i$ at time $T_{l-1}$ to $X(T_{l})=n$, $J(T_{l})=j$ at time $T_{l}$ happens with probability

(33) $$\int_{0}^{\infty }\frac{e^{-\mu t}{\left(\mu t\right)}^{k+1-n}}{(k+1-n)!}f_{i,j}\left(t\right)dt,$$

if 0<n≤k+1≤K and with probability

(34) $$\sum_{u=k+1}^{\infty }\int_{0}^{\infty }\frac{e^{-\mu t}{\left(\mu t\right)}^{u}}{u!}f_{i,j}\left(t\right)dt,$$

if n=0 and k+1≤K. Fourth, the packet arriving at $T_{l}$ must be rejected, to initiate a new series of losses. This happens with probability d(n). Defining $h_{n,j}$ to be the probability that a series of losses begins at an arrival time in the stationary regime when the queue size is *n* and the modulating state is *j*, we have, therefore,

(35) $$h_{0,j}=\sum_{i=1}^{m}\sum_{k=0}^{K-1}\pi_{k,i}\left(1-d(k)\right)d\left(0\right)\sum_{u=k+1}^{\infty }\int_{0}^{\infty }\frac{e^{-\mu t}{\left(\mu t\right)}^{u}}{u!}f_{i,j}\left(t\right)dt,$$

(36) $$h_{n,j}=\sum_{i=1}^{m}\sum_{k=n-1}^{K-1}\pi_{k,i}\left(1-d(k)\right)d\left(n\right)\int_{0}^{\infty }\frac{e^{-\mu t}{\left(\mu t\right)}^{k+1-n}}{(k+1-n)!}f_{i,j}\left(t\right)dt,\;\;\;n=1,\ldots ,K.$$

Denote

(37) $$h_{n}=\left[h_{n,1},\ldots ,h_{n,m}\right].$$

(35) and (36) yield

(38) $$h_{0}=d\left(0\right)\sum_{k=0}^{K-1}\left(1-d(k)\right)\pi_{k}\left({\left(\Lambda -Q\right)}^{-1}\Lambda -\sum_{u=0}^{k}M_{u}\right),$$

(39) $$h_{n}=d\left(n\right)\sum_{k=n-1}^{K-1}\left(1-d(k)\right)\pi_{k}M_{k+1-n},$$

respectively.

To compute $\pi_{n,i}$, consider a long time interval of length $\bar{t}$. In this interval, there are $\lambda \bar{t}$ arrivals in total, while $\lambda_{i}p_{n,i}\bar{t}$ arrivals when the queue size is *n*, and the modulating state is *i*. Therefore, the probability that, upon arrival, the queue size is *n* and the modulating state is *i* must be $\lambda_{i}p_{n,i}\bar{t}/\left(\lambda \bar{t}\right)=\lambda_{i}p_{n,i}/\lambda$. Hence, we have

(40) $$\pi_{n}=\frac{1}{\lambda }p_{n}\Lambda ,$$

where λ and $p_{k}$ are given in (10) and (8), respectively.

Combining (40) with (38) and (39), we obtain (14) and (15), respectively.

The last missing component needed to compute *B* by means of (2) is the loss ratio, *L*. Considering an arbitrary arrival epoch in the stationary regime, we see that it must be

(41) $$L=\sum_{n=0}^{K}d\left(n\right)\pi_{n}e.$$

From (41) and (40), we obtain (17).

Finally, note that the probability that, at an arbitrary arrival time, a series of losses is initiated is equal to $\sum_{n=0}^{K}h_{n}e$. On the other hand, the mean length of the series of losses, under the condition that it begins when the queue size is *n*, is equal to $h_{n}E_{n}$. Therefore, the mean length of a series that starts at an arbitrary time, no matter what the queue size is, must be equal to $\sum_{n=0}^{K}h_{n}E_{n}/\sum_{n=0}^{K}h_{n}e$. This, combined with Formula (2), yields (11), and the proof is completed.    □

## 5. Numerical Examples

In these examples, rather than using one particular arrival process, we used a family of processes, dependent on a parameter q>0. Namely, a member of family $MMPP_{q}$ is parameterized as follows:

(42) $$Q=\frac{1}{q}\cdot \left[\begin{array}{ccc} -0.03746 & 0.02241 & 0.01505\\ 0.01565 & -0.03319 & 0.01754\\ 0.02430 & 0.01003 & -0.03433 \end{array}\right],$$

(43) Λ=diag[0.03594,0.21831,2.84660].

**Figure 1:**
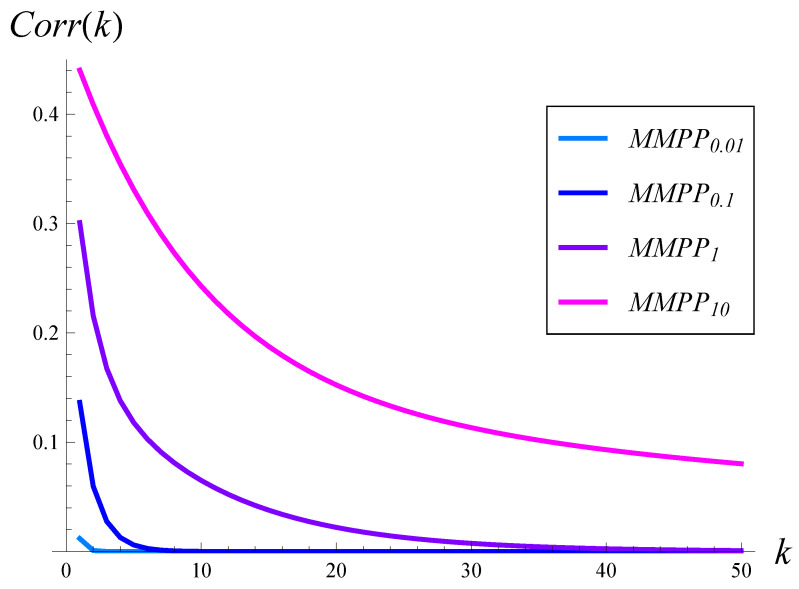
Autocorrelation of interarrival times vs. lag for different values of parameter *q*.

These matrices were selected so that, no matter what the value of *q* is, the total arrival rate is λ=1. Parameter *q* is meant to set the autocorrelation of the interarrival times. Namely, the larger *q* is, the stronger and longer-range autocorrelation is. Roughly speaking, the autocorrelation is practically negligible for q=0.01, mild for q=0.1, moderate for q=1, and very strong for q=10. The autocorrelation function for a few selected values of *q* is depicted in Figure 1. Manipulating *q*, we were able to study the dependence of the burst ratio on the autocorrelation of the arrival process, without altering the link load.

We also used a parameter-dependent family, $d_{r}$, of drop probability functions:

(44) $$d_{r}\left(n\right)=\left\{\begin{array}{ccc} 0, & \mathrm{if} & n<32,\\ {\left(\frac{n-32}{32}\right)}^{r}, & \mathrm{if} & 32\leq n<64,\\ 1, & \mathrm{if} & n\geq 64, \end{array}\right.$$

In (44), *r* is some positive parameter, while the buffer size is K=64 packets. Up to 50 percent occupancy of the buffer, there is no dropping: it begins when the occupancy exceeds 50 percent. Using *r*, we can control the strength of the drop probability function, i.e., the smaller *r* is, the stronger the dropping is. For instance, for r=0.5, the function is the square root, for r=1 linear, for r=2 quadratic, for r=3 cubic, etc. Manipulating *r*, we were able to study the dependence of the burst ratio on the strength of the drop probability function.

The same buffer size, 64, was used in the examples when AQM was not applied at all. In such a case, packets were dropped due to buffer overflows.

Finally, the service rate was used to control the load. As we normalized the arrival rate, λ=1, the load is simply a reciprocal of the service rate, ρ=1/μ.

### 5.1. Dependence on Autocorrelation

**Figure 2:**
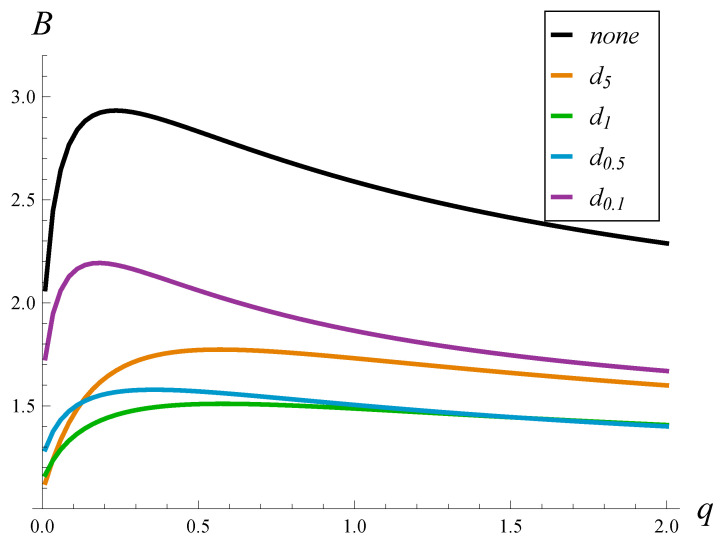
Burst ratio vs. the autocorrelation strength for a few functions d(n) and no AQM. Link load ρ=0.8.

**Figure 3:**
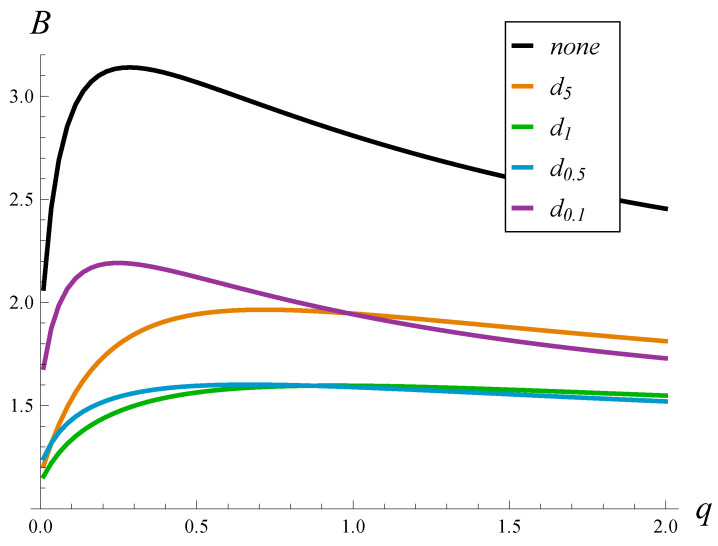
Burst ratio vs. the autocorrelation strength for a few functions d(n) and no AQM. Link load ρ=1.2.

We firstly checked the influence of the strength of the autocorrelation on the burst ratio. In Figure 2 and Figure 3, the burst ratio versus *q* is depicted for a few drop probability functions and the lack thereof. Figure 2 was obtained for an underloaded link, ρ=0.8, while Figure 3 for an overloaded link, ρ=1.2.

We can notice a few interesting things in Figure 2 and Figure 3. First of all, the burst ratio in the case with no AQM is always significantly higher than in cases with AQM and reaches sometimes very high values, around 3. An application of function $d_{r}$ reduces *B* substantially. Among the considered functions $d_{r}$, the best results were obtained for $d_{1}$ and $d_{0.5}$ and worse for $d_{5}$ and $d_{0.1}$. This means that we need to balance the drop probabilities: too high or too low are not optimal. This effect is studied further in Section 5.2.

Perhaps the most-interesting observation in Figure 2 and Figure 3 is that the burst ratio does not grow monotonically with the strength of the autocorrelation. In every case, there is clearly a maximum for a moderate autocorrelation, i.e., for *q* around 0.2–0.5. This is rather bad news from the networking perspective, where we can expect the strength of the autocorrelation to be just like this, i.e., neither very weak nor extremely strong.

Note that such a non-monotonic behavior of the burst ratio is different than the behavior of other queueing characteristics (e.g., the loss ratio, the queue size), which are known to grow with the strength of the autocorrelation.

### 5.2. Dependence on Drop Probabilities

Now, we can check the influence of the drop probabilities on the burst ratio.

**Figure 4:**
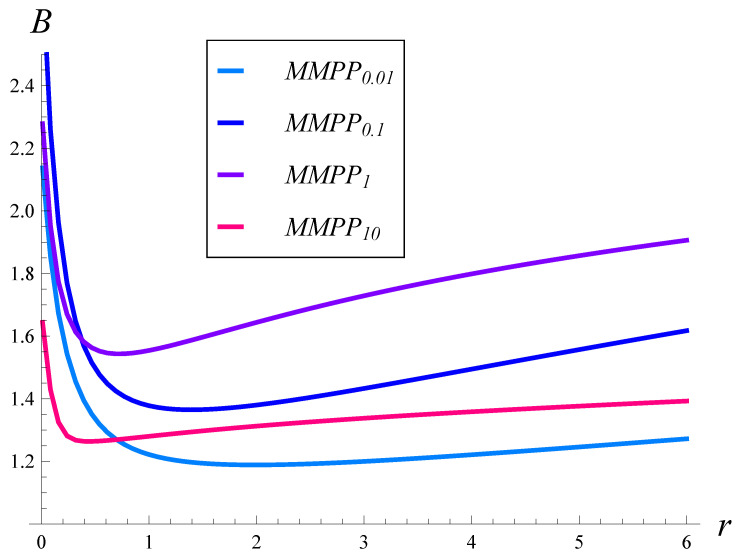
Burst ratio vs. the strength of the drop probability function for arrival processes with different autocorrelations. Link load ρ=1.0.

In Figure 4, the burst ratio versus parameter *r* of function $d_{r}\left(n\right)$ is depicted for a few arrival processes, i.e., $MMPP_{0.01}$, $MMPP_{0.1}$, $MMPP_{1}$, and $MMPP_{10}$. A load of 1 was assumed in every case. (When examining Figure 4, we should be reminded that the strength of the drop probability function decreases with *r*, i.e., a larger *r* means a weaker $d_{r}$.)

As we can see in Figure 4, for every arrival process, there is an optimal value of *r*, for which the burst ratio reaches its minimum. This optimal value, however, can be quite different for arrival processes of different autocorrelations. The optimal *r* is about 2 for $MMPP_{0.01}$, about 1.3 for $MMPP_{0.1}$, about 0.7 for $MMPP_{1}$, and about 0.4 for $MMPP_{10}$. Hence, when the autocorrelation becomes stronger, a stronger drop probability function is required to achieve the best-possible burst ratio.

From this observation, it clearly follows that it is not possible to have one, universal function $d_{r}$ that provides the best burst ratio in all networking conditions. One value of *r* may provide an optimal *B* for a particular traffic autocorrelation, but when this autocorrelation changes, the resulting *B* may be far from optimal.

### 5.3. Dependence on the Link Load

Now, we can check the influence of the load of the link on the burst ratio.

**Figure 5:**
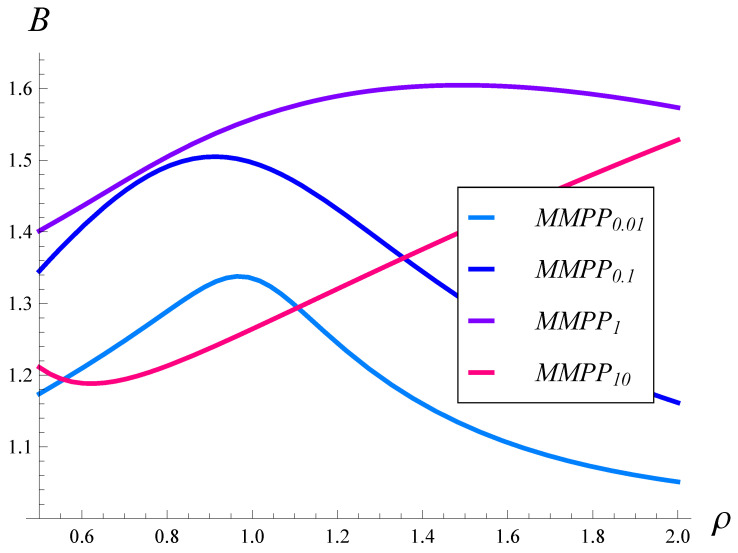
Burst ratio vs. the link load for arrival processes with different autocorrelations. Square root drop probabilities, i.e., r=0.5.

**Figure 6:**
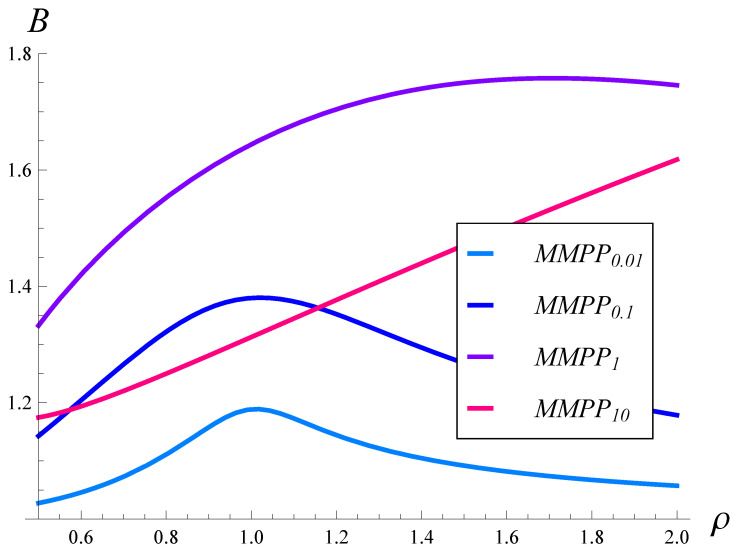
Burst ratio vs. the link load for arrival processes with different autocorrelations. Quadratic drop probabilities, i.e., r=2.

In Figure 5 and Figure 6, the burst ratio versus ρ is depicted for the square root and quadratic drop probability functions, respectively. In both figures, the results for different arrival processes, $MMPP_{0.01}$, $MMPP_{0.1}$, $MMPP_{1}$, and $MMPP_{10}$, are depicted.

As can be seen, the burst ratio as a function of ρ is non-monotonic in most cases. Moreover, the autocorrelation has a profound effect on the dependence. The burst ratio reaches a maximum for a ρ of about 1, when the autocorrelation is low ($MMPP_{0.01}$ and $MMPP_{0.1}$), and for a ρ of about 1.5, when the autocorrelation is moderate ($MMPP_{1}$). Most likely, there is also a maximum for $MMPP_{10}$, but for a very high ρ. There is also a minimum in Figure 5 for $MMPP_{10}$.

When comparing Figure 5 and Figure 6 with each other, we see that the quadratic drop function performed slightly better when the autocorrelation was low. On the other hand, when the autocorrelation became moderate, the square root function prevailed slightly. In general, the respective curves are similar between Figure 5 and Figure 6, and the maxima are located in similar places. This means that the impact of the drop probability function was less than the impact of the autocorrelation, which makes the curves within each figure significantly different.

### 5.4. Simulations

We also performed simulations to check the theoretical results for possible errors. For this purpose, we implemented, faithfully, in a simulator, the system defined in Section 3.

The traffic was simulated according to the MMPP matrices *Q* and Λ given in (42) and (43), respectively. To all the packets arriving to the queue, the dropping scheme with the function $d_{r}\left(n\right)$ given in (44) was applied. Each simulation lasted as long as some predefined number of packets passing through the simulated system. During this time, the burst ratio parameter was measured.

The simulation of the MMPP can be performed using quantities $b_{i}$ and $q_{i,j,k}$ defined as follows:

(45) $$b_{i}=-{\left(Q-\Lambda \right)}_{ii},\;\;\;\;\;\;\;\;i=1,\ldots ,m,$$

(46) $$q_{i,i,0}=0,\;\;\;\;\;\;\;i=1,\ldots ,m,$$

(47) $$q_{i,j,0}=\frac{1}{b_{i}}{\left(Q-\Lambda \right)}_{ij},\;\;\;\;\;\;\;i,j=1,\ldots ,m,\;\;\;i\neq j,$$

(48) $$q_{i,j,1}=\frac{1}{b_{i}}{\left(\Lambda \right)}_{ij},\;\;\;\;\;\;\;i,j=1,\ldots ,m,$$

where ${\left(A\right)}_{ij}$ denotes the (i,j)-thentry of matrix *A*.

Having $b_{i}$ and $q_{i,j,k}$, we can describe the progression of an MMPP in the following way. Given that the modulating Markov chain is in state *i*, it remains in this state for a random time, exponentially distributed with parameter $b_{i}$. At the end of this time, the state of the Markov chain changes to *j*, which happens with probability $q_{i,j,0}$, or a new packet arrives, which happens with probability $q_{i,i,1}$. The simultaneous change of the modulating state and a packet arrival is impossible, due to the fact that $q_{i,j,1}=0$ if i≠j. Then, the procedure repeats, with the same or a new (if changed) modulating state.

In Algorithm 1, a practical realization of this procedure is presented in pseudocode. It produces the next packet interarrival time and updates the state of the modulating Markov chain. In this code, *i* is an external variable keeping the state of the modulating chain, *m* is the number of states of this chain, uniform(0,1) is a pseudorandom number from interval (0,1), arrays q[][][] and b[] store numbers $q_{i,j,k}$ and $b_{i}$ defined above, while the logarithm is used to generate an exponentially distributed random variable.
**Algorithm 1** MMPP interarrival time generation and state update. t←0; **do**     s←0;     r←uniform(0,1);     **for** k=0,1 **do**         **for** j=1,…,m **do**            s←s+q[i][j][k];            **if** s>r **then**                break;         **if** s>r **then**            break;     t←t−log(1−uniform(0,1))/b[i];     i←j;                /*updates the modulating state*/ **while**
k=0; return
*t*;                 /*returns the next interarrival time*/ 

The simulations were implemented and performed in the modular simulator OMNeT++ [43]. Several different configurations of the system were tested in the simulator, with different combinations of the autocorrelation, drop probabilities (and the lack thereof), and link loads. In every run of the simulator, 100 million packets passing through the queue were simulated.

Sample results are gathered in Table 1. As we can see, in every case, the burst ratio obtained in the simulation conformed very well with the burst ratio obtained via Theorem 1.

## 6. Conclusions

We showed, using a new theorem, that AQM algorithms based on the queue size can reduce the burst ratio parameter significantly. This is an additional nice feature of them, because they were not designed with the burst ratio in mind.

To show this, we derived a formula for the burst ratio in a model of the system enabling an arbitrary autocorrelation function and an arbitrary interarrival time distribution of the traffic. Using this formula, several numerical examples were presented, in which the influence of different system parameters on the burst ratio was presented.

Firstly, we focused on the influence of the autocorrelation, which was an important feature of the considered model. The most-interesting observation was that the burst ratio did not grow monotonically with the strength of the autocorrelation. Such behavior of *B* is different than the behavior of other queueing characteristics (the loss ratio, the queue size), which are known to grow with the strength of the autocorrelation. In the considered examples, the burst ratio reached its maximum for a moderate strength of the autocorrelation. This is rather bad news from the networking perspective, where we can expect the strength of the autocorrelation to be just like this.

An application of AQM significantly reduced the burst ratio in every case. We considered a parameter-dependent family of drop probability functions and singled out the optimal function with respect to *B*, for every considered traffic parametrization. Apparently, the form of the optimal function depends strongly on the traffic autocorrelation, in a quasi-monotonic manner: the stronger the autocorrelation is, the stronger the drop probability function needed to achieve the best burst ratio.

## Figures and Tables

**Table 1:** Theoretical vs. simulated burst ratio for different system parameters (arrival process, drop probabilities, and link load).

System	Theor.	Simul.
Parameters	*B*	*B*
$MMPP_{1}$, no AQM, ρ=1.0	2.7129	2.7141
$MMPP_{1}$, $d_{5}$, ρ=0.7	1.6505	1.6506
$MMPP_{1}$, $d_{2}$, ρ=1.0	1.6441	1.6443
$MMPP_{1}$, $d_{0.5}$, ρ=1.3	1.5984	1.5983
$MMPP_{0.1}$, no AQM, ρ=1.2	2.9162	2.9169
$MMPP_{0.1}$, $d_{2}$, ρ=0.9	1.3626	1.3628
$MMPP_{0.1}$, $d_{1}$, ρ=1.2	1.3351	1.3349
$MMPP_{0.1}$,$d_{0.25}$, ρ=1.5	1.4938	1.4939
$MMPP_{0.01}$, no AQM, ρ=1.5	1.8583	1.8578
$MMPP_{0.01}$, $d_{1}$, ρ=1.0	1.2220	1.2218
$MMPP_{0.01}$, $d_{0.5}$, ρ=1.5	1.1298	1.1298
$MMPP_{0.01}$, $d_{0.25}$, ρ=2.0	1.1207	1.1206

## Data Availability

Not applicable.
